# A case of pneumomediastinum complicating immunotherapy associated pneumonitis

**DOI:** 10.1002/rcr2.1406

**Published:** 2024-06-17

**Authors:** Anthony Carrozzi, Rex Chu, William Nigole, Hariette Goldman, Serena Hope, Richard Li, Con Archis

**Affiliations:** ^1^ Respiratory Medicine The Sutherland Hospital Sydney New South Wales Australia

**Keywords:** bronchoscopy, immunotherapy, pneumomediastinum, pneumonitis

## Abstract

We describe the case of an 87‐year‐old gentleman referred to a metropolitan hospital in Sydney with pneumomediastinum complicating immunotherapy associated pneumonitis and recent bronchoscopic intervention. The contribution of pneumonitis in the setting of interstitial lung disease has been well described to developing pneumomediastinum however this is less clear in the setting of immunotherapy associated pneumonitis and to what extent bronchoscopic intervention compounds this risk.

## Graphical abstract

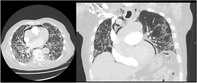

## INTRODUCTION

Pneumomediastinum is a rare condition which can be either primary/spontaneous or secondary. Spontaneous pneumomediastinum is commonly associated with younger males of tall stature as opposed to secondary pneumomediastinum which is primarily associated with rapid changes in intra‐thoracic pressure.^1^ Both interstitial lung diseases (ILD) and bronchoscopic procedures are well described as possible secondary causes of pneumomediastinum with reported incidence rates of up to 4% on transbronchial biopsy and 2% on endobronchoscopic ultrasound (EBUS) guided biopsy.^2^, ^3^, ^4^ Bronchoscopy is frequently performed in the investigation of immunotherapy associated pneumonitis to exclude other aetiologies including infection. However the risk of pneumomediastinum from bronchoscopic evaluation of active pneumonitis has not been well elucidated in the literature.^5^

## CASE REPORT

An 87‐year‐old lifelong non‐smoker presented to a metropolitan hospital in Sydney in January 2024 with exertional dyspnoea for evaluation. History was significant for MSI high, BRAF positive bowel cancer managed with a right hemicolectomy in October 2021 and adjuvant, maintenance pembrolizumab immunotherapy. Treatment was complicated by immunotherapy associated colitis confirmed in December 2023. Pembrolizumab was withheld in August 2023 and treated with a tapering course of prednisolone that concluded prior to presentation. Other history includes right scapula radiotherapy in November 2022, and unprovoked pulmonary embolism.

The exertional dyspnoea was investigated with clinical, pathological and radiographic modalities including CT pulmonary angiography which excluded recurrent pulmonary embolism but identified diffuse ground glass opacities. The patient was treated for community acquired pneumonia with intravenous ceftriaxone and oral doxycycline. Relapse of an immunotherapy related adverse event (IRAE) was considered and the patient was treated with 1 mg/kg of oral prednisolone empirically. A bronchoscopy was performed, however the procedure was abandoned prior to the completion of procedure due to desaturation. Within the limits of the procedure, no endobronchial lesions were identified. Washings of the right upper and middle lobes were negative for an infectious aetiology and antibiotic therapy was ceased. The patient was extensively investigated for an infectious cause of the ground glass changes in the context of preceding immunosuppression, specifically testing for COVID‐19, Influenza, RSV, Adenovirus, Enterovirus, RSV, Parainfluenzae, Bocavirus, Cryptococcus, Aspergillus, Cytomegalovirus, Legionella, Mycoplasma and Tuberculosis in addition to the bacterial and fungal cultures on bronchial washings. An autoimmune, rheumatological cause was felt to be less likely in the setting of recent immunosuppression with corticosteroids. In the setting of a normal ESR and CRP the patient was not evaluated any further for autoimmune aetiologies.

**FIGURE 1:**
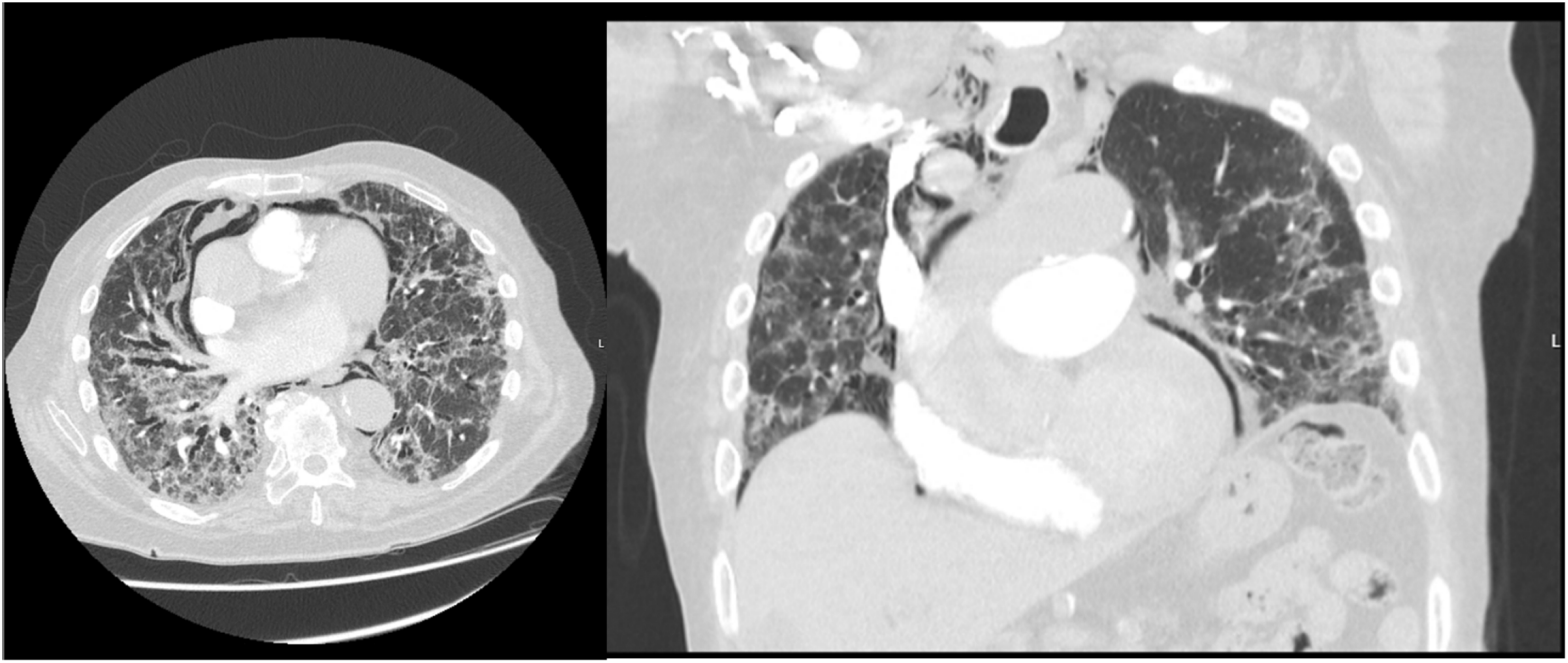
CT pulmonary angiogram demonstrating pneumomediastinum.

The patient was monitored as an inpatient for the following 5 days and slowly weaned off oxygen to room air. Despite ongoing exertional desaturation events, the patient was subsequently discharged against medical advice due to personal/social concerns. He was provided a weaning course of oral prednisolone. On repeat CT chest prior to outpatient oncology follow up, pneumomediastinum was identified and the patient was admitted for investigation and management, see Figure 1. Besides ongoing exertional dyspnoea the patient remained asymptomatic despite this finding and was saturating 95% on room air. Alternative causes for pneumomediastinum were excluded. The patient was managed with analgesia, cough suppressants and supplemental oxygen as per consensus guidelines (as per secondary pneumothorax management). The patient remained stable and was discharged with oxygen at home and planned for close respiratory and oncology follow up.

## DISCUSSION

**FIGURE 2:**
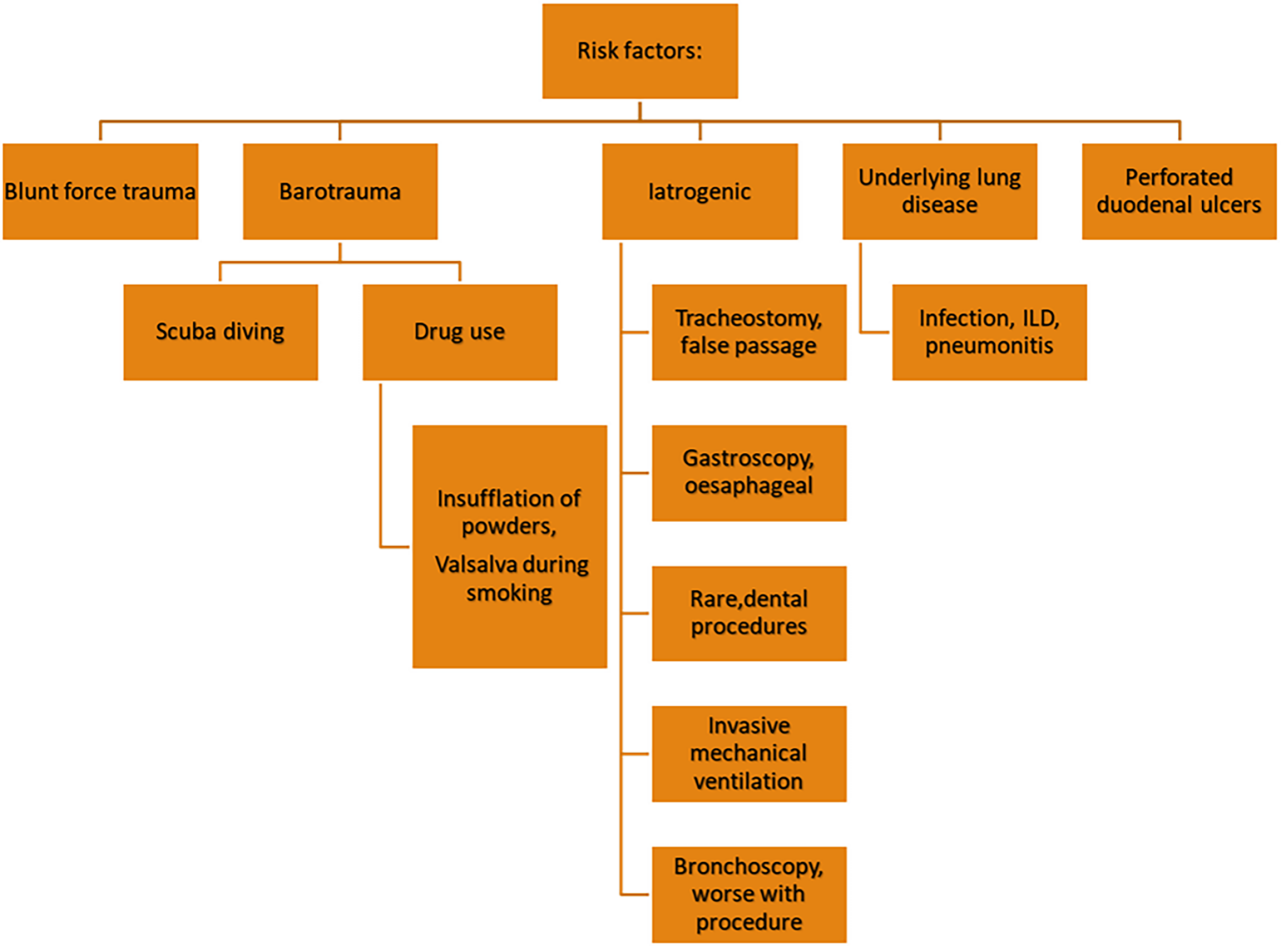
Described aetiologies of secondary pneumomediastinum.

Pneumomediastinum has been well described as a complication of active pneumonitis in the setting of ILD and additionally as a rare complication of bronchoscopy, although primarily related to interventions such as transbronchial or EBUS biopsy.^3^ In the setting of ILD it is well understood that active inflammation in combination with architectural distortion increases the propensity of alveolar rupture which causes air‐leak into the mediastinal space, see Figure 2. This then dissects distally producing signs and symptoms such as chest pain, dyspnoea, subcutaneous emphysema and, in extreme cases, tension pneumomediastinum and circulatory collapse.^1^ A similar pathophysiological process occurs in transbronchial and EBUS biopsy which can create an iatrogenic air‐leak into the mediastinal space.^3^ This case describes a common clinical conundrum of differentiating IRAE associated pneumonitis from an infectious process in the setting of recent prednisolone. The authors postulate that primarily active IRAE pneumonitis was the most likely source of the development of pneumomediastinum. In this particular case, the role of prolonged course of corticosteroids of IRAE colitis may have contributed to tissue friability which may have predisposed to development and persistent of pneumomediastinum. This could be in part related to positive end expiratory pressure associated with high flow nasal prong oxygen therapy. To the knowledge of the authors, pneumomediastinum has not been well described as a complication of IRAE pneumonitis previously.

## AUTHOR CONTRIBUTIONS

All authors were involved directly in either the care of the patient in this case and/or authorship of this manuscript.

## CONFLICT OF INTEREST STATEMENT

None declared.

## ETHICS STATEMENT

The authors declare that appropriate written informed consent was obtained for the publication of this manuscript and accompanying images.

## Data Availability

No data are available.
